# Advances in the Characterization of the T-Cell Response to Human Herpesvirus-6

**DOI:** 10.3389/fimmu.2018.01454

**Published:** 2018-06-25

**Authors:** Derek J. Hanson, Joshua A. Hill, David M. Koelle

**Affiliations:** ^1^Department of Medicine, University of Washington, Seattle, WA, United States; ^2^Vaccine and Infectious Disease Division, Fred Hutchinson Cancer Research Center, Seattle, WA, United States; ^3^Department of Laboratory Medicine, University of Washington, Seattle, WA, United States; ^4^Department of Global Health, University of Washington, Seattle, WA, United States; ^5^Benaroya Research Institute, Seattle, WA, United States

**Keywords:** human herpesvirus 6, CD4+ T cell, CD8+ T cell, antigen, epitope

## Abstract

Human herpesvirus (HHV) 6 is thought to remain clinically latent in most individuals after primary infection and to reactivate to cause disease in persons with severe immunosuppression. In allogeneic hematopoietic stem cell transplant recipients, reactivation of HHV-6 species B is a considerable cause of morbidity and mortality. HHV-6B reactivation is the most frequent cause of infectious meningoencephalitis in this setting and has been associated with a variety of other complications such as graft rejection and acute graft versus host disease. This has inspired efforts to develop HHV-6-targeted immunotherapies. Basic knowledge of HHV-6-specific adaptive immunity is crucial for these endeavors, but remains incomplete. Many studies have focused on specific HHV-6 antigens extrapolated from research on human cytomegalovirus, a genetically related betaherpesvirus. Challenges to the study of HHV-6-specific T-cell immunity include the very low frequency of HHV-6-specific memory T cells in chronically infected humans, the large genome size of HHV-6, and the lack of an animal model. This review will focus on emerging techniques and methodological improvements that are beginning to overcome these barriers. Population-prevalent antigens are now becoming clear for the CD4+ T-cell response, while definition and ranking of CD8+ T-cell antigens and epitopes is at an earlier stage. This review will discuss current knowledge of the T-cell response to HHV-6, new research approaches, and translation to clinical practice.

## Introduction

Human herpesvirus 6 was discovered in 1986 and later found to exist as two closely related species, HHV-6A and HHV-6B, in the *Betaherpesvirinae* subfamily and *Roseolovirus* genus (1). Hereafter, “HHV-6” refers to both species unless specific data are available to differentiate between species. The two species have genomes roughly 162 kb long with 88–90% sequence identity, but have distinct tropisms and epidemiology (1). The other betaherpesviruses known to infect humans are HHV-7 and human cytomegalovirus (HCMV). About 1% of humans have inherited chromosomally integrated HHV-6 (ici-HHV-6) (2). Interestingly, immune tolerance has not been demonstrated and persons with ici-HHV-6 appear to maintain anti-HHV-6 cell-mediated immunity (CMI) (3). Primary infection with HHV-6B usually occurs once maternal antibodies have waned in early life (4, 5). The clinical syndrome roseola consists of fever and rash, although seizures can occur. The epidemiology of HHV-6A is less well understood, related to difficulties with species-specific serodiagnosis. Like other herpesviruses, HHV-6 establishes lifelong latent infection, usually asymptomatic. Transmission is probably *via* saliva, as HHV-6 DNA is frequently detectable in oral specimens.

Human herpesvirus-6 reactivation events are thought to occur periodically in healthy carriers and to be subclinical due to intact immune surveillance. Natural killer cells appear to have anti-HHV-6 function (6), as implied by their activity in the acute febrile phase of primary infection (7, 8) and cytotoxicity against HHV-6-infected cells (9) in an interleukin-15-dependent manner (10). There is little evidence that antibody deficiency disorders increase risk of complications from infection by these viruses (11), and B cell deficiency does not increase lethality of murine roseolovirus (MRV), a betaherpesvirus related to HHV-6, in neonatal mice (12).

Compared to other herpesviruses, HHV-6-specific cell-mediated response is delayed in primary infection (8). This correlates with, and could be mechanistically related to, HHV-6 lymphotropism (13–15), since activated HHV-6-responsive T cells may be differentially susceptible to destructive viral infection. HHV-6 also has immunosuppressive mechanisms targeting T cell function (16–20). The T-cell response is considered critical for control of HHV-6B infection since reactivation commonly occurs in cases of T-cell lymphopenia, e.g., in AIDS patients (21) or after bone marrow transplantation (22–28). Moreover, greater overall survival in these posttransplant patients is associated with at least 200 CD3+ T cells/μL in blood at the time of HHV-6B reactivation (29).

The relative importance of different T-cell subsets in HHV-6B immunity is still not well established. In pediatric hematopoietic cell transplant (HCT) patients, increased proportions of perforin-expressing CD8+ T cells have been temporally associated with HHV-6 clearance (30). HHV-6-specific CD8+ T cells with proliferative capacity were more readily detectable in patients after viral reactivation but not in those without (31). Moreover, MRV is lethal to CD8 knockout mice but not to wild-type mice (12). Nevertheless, like other herpesviruses (32–38), HHV-6 can evade CD8+ T cells by downregulating class I MHC molecules (39), which may account for challenges in detecting HHV-6B-specific CD8+ T cells (40, 41).

CD4+ T cells are now considered to exert their own direct antiviral effector functions and to be crucial in controlling herpesvirus infections (42–47), although less is known about their importance for HHV-6B control. Some observers consider it plausible that HHV-6B-induced *de novo* surface expression of class II MHC molecules (48)—similar to HCMV (49, 50)—could promote recognition of infected cells by CD4+ T lymphocytes. Moreover, HHV-6A-specific CD4+ T-cell lines can produce IFNγ and degranulate (measured by surface CD107a/b) when presented with whole virus or peptide antigen, suggesting HHV-6A-specific cytotoxicity (51). These *in vitro* studies suggest the importance of Th1 cytotoxic CD4+ T cells in immunity to HHV-6. However, lack of an animal model, the multifaceted nature of human immunodeficiency states such as transplantation and HIV infection, and a paucity of data from direct *ex vivo* methods to measure expression of cytotoxic machinery in HHV-6-specific CD4+ T cells precludes strong conclusions at present.

## Medical Importance of HHV-6

The strongest evidence supporting clinically significant consequences of HHV-6 infection is in immunocompromised patients, particularly recipients of allogeneic HCT and solid organ transplants (SOT). Detection of HHV-6B DNA in blood occurs in 40–50% of HCT recipients at a median of approximately 3 weeks after transplant, corresponding with the time period of lowest lymphocyte and neutrophil counts (25–28, 52, 53). HHV-6B accounts for ≥98% of HHV-6 detection after allogeneic HCT. Given pre-existing seropositivity, most viral detection is likely due to viral reactivation. The factors most prominently associated with HHV-6B reactivation include the use of umbilical cord blood stem cells as the donor source, an HLA mismatched or unrelated donor, receipt of depleting anti-T-cell antibodies, development of acute graft-versus-host disease, and treatment with glucocorticoids (26, 28, 54–59).

Human herpesvirus-6B is the most frequent infectious cause of encephalitis after allogeneic HCT, and this occurs in approximately 1% of all HCT recipients (26, 27, 60–62). HHV-6B encephalitis results in significant morbidity and mortality despite antiviral treatment (26, 61, 63, 64). HHV-6B reactivation has been detected in many other conditions in allogeneic HCT and SOT recipients, although its causal role is less clearly defined. These include myelosuppression, development of acute GVHD (26, 28, 65, 66), increased risk for cytomegalovirus reactivation and disease (28, 67–71), and solid organ allograft dysfunction (72–74).

Several available antiviral agents demonstrate activity against HHV-6A and HHV-6B, including foscarnet, ganciclovir, and cidofovir (25, 75–78), but clinical use has been limited by the toxicities of available antiviral agents and lack of proven efficacy in preventing end-organ disease (79–81). New small molecules with activity against HHV-6 species are in development (77, 82, 83). Given these shortcomings, adoptive immunotherapy using virus-specific T cells (VSTs) is an exciting new therapeutic approach that appears to be safe and to reduce HHV-6 DNA levels, as well as end-organ disease symptoms, in small, uncontrolled studies (84–87). More research is needed to identify HHV-6 epitopes that can be used to generate high affinity T-cell lines to advance adoptive immunotherapeutic strategies.

## Challenges and Opportunities for T-Cell Epitope Discovery in HHV-6

Several technical challenges confront investigators seeking to identify T-cell epitopes in HHV-6 and study the roles of T cells in pathogenesis and immunity. First, non-infected humans to serve as negative controls are rare. Second, an affordable small animal model for HHV-6 infection is not available for *in vivo* studies. Pig tailed macaques (88), marmosets (89), cynomolgus macaques, and African green monkeys (90) are susceptible to human roseolovirus strains and have been used as models in a few studies (91, 92). Endogenous roseoloviruses have recently been described in chimpanzees (93) and rhesus macaques (94). Once we understand more about the natural history of these infections in their natural hosts and how well they mimic HHV-6 in humans, it may become rational to use them as informative model systems. Recently, the genomic sequence of a mouse herpesvirus, MRV, was revealed and appears phylogenetically closer to HHV-6 than to murine cytomegalovirus. It is considered a homolog of HHV-6 and HHV-7 with some potential as an *in vivo* model (95).

T-cell antigen identification and ranking in animals using animal homologs of HHV-6 may not translate to the HHV-6 infection setting in humans; the optimal human specimens for understanding the *in vivo* reality have not been defined. Samples from immunocompetent humans with primary or reactivated HHV-6 infection (ideally taken at the peak of virus-specific T cell expansion) might have a high enough frequency of HHV-6-specific T cells to allow direct *ex vivo* analysis of antigen specificity. This could provide the most true-to-life ranking of HHV-6 antigens with regard to population prevalence and within-patient immunodominance. Samples from such patients have been used in studies of HHV-6 viral loads and clinical associations (96–99), but we are not aware of their use for T cell antigen discovery so far. In addition, study designs in which serial blood samples are obtained from patients with primary or reactivated HHV-6B who spontaneously control infection may help to understand the effective versus bystander T-cell responses. Identification of HHV-6B antigens that activate T cells in this setting may give clues as to the nature of an effective T cell response.

Third, HHV-6A and -6B are quite evasive, with mechanisms including downregulation of CD3 (88), induction of interleukin-10-producing T-regulatory type 1 cells (18), and inhibition of interferon beta (19), interleukin 2 (20), interleukin 12 (100, 101), and MHC class I (6, 39, 102, 103). Perhaps this is why HHV-6-specific T cells in healthy persons, as measured by IFNγ production, are quite rare; between 0.01 and 0.1% of CD4+ T cells respond to whole virus (51) while CD8+ T cells specific for HHV-6 peptide-loaded pentamers are often less than 0.01% of circulating CD8+ T cells and sometimes below the limit of detection (104, 105). The overall frequency of CD8+ T cells specific for whole HHV-6 virus has not been established. By contrast, up to 20% of memory CD4 and CD8 T cells are specific for HCMV in seropositive persons (106). *Ex vivo* testing of HHV-6B CD4+ T-cell abundance in PBMC by standard methods, such as ELISPOT or intracellular cytokine secretion (ICS), is reliably quantitative if enough cells are studied, but not sensitive enough to meaningfully characterize fine epitope specificity. Researchers, therefore, use *in vitro* expansion to enrich HHV-6-reactive T cells for detailed epitope identification and definition of epitope breadth and population prevalence (40, 41, 51, 104, 105, 107, 108). However, this process may introduce changes in T-cell expression profiles, and differential proliferation rates could result in skewed T-cell clonotype proportions in the expanded product, introducing challenges into the larger goals of studying the phenotype of HHV-6-specific T cells and measuring the immunodominance hierarchy across a spectrum of epitopes. Overall, *in vitro* expansion of VSTs offers enhanced sensitivity but decreased quantitative precision (Table 1).

**Table 1:** Advantages and disadvantages of approaches to HHV-6 epitope discovery.

Approach	Advantages	Disadvantages
Selected proteins based on human cytomegalovirus homology	Enables scanning for epitopes in reasonable blood volumes from persons with diverse HLA types	Leaves most HHV-6 proteins unexplored for epitopes

Epitope prediction based on selected HLA restrictions	Provides an efficient method to scan entire viral proteome space for epitopes	HLA-binding affinity alone is an inconsistent predictor of actual immunogenicity
		Leaves unexplored epitopes recognized by other HLA alleles

*Ex vivo* T cell responders	High precision: relative abundances and phenotype closely approximate *in vivo* biology	Low sensitivity: HHV-6-specific T cells are rare and frequently below the lower limit of detection

*In vitro* expanded T cell responders	High sensitivity: can detect infrequent T-cell specificities and define a detailed hierarchy of population prevalence	Low precision: expansion process could skew proportions of T-cell clonotypes and/or change their gene expression profiles

Finally, the HHV-6B genome contains roughly 100 open reading frames totaling tens of thousands of potential T-cell epitopes—a large potential epitope space. Investigators are faced with either down-selecting to a limited number of open reading frames, or using high-throughput methods requiring considerable time and expense.

## Approaches to T-Cell Epitope Discovery in HHV-6

Approaches to discovery of T-cell epitopes in HHV-6B can be categorized by the methods they use to address these challenges. One system focuses on selected HHV-6B proteins and scans cells from persons with a range of HLA haplotypes for induction of T-cell activation. Using the better-characterized HCMV as a springboard, early studies of HHV-6B focused on proteins, which have HCMV homologs (17, 40, 104, 105). The rationale is not possible cross-reactivity due to homolog sequence identity; for example, the identity of U54 to its HCMV homolog UL83 (encoding pp65) is only 20% (104). Rather, it was hypothesized that T-cell antigenicity may be related to biological functional, viral mRNA and protein expression kinetics, virion abundance, and other factors, predisposing certain betaherpesvirus proteins to higher antigenicity.

A second approach is to focus on HHV-6B peptides predicted to bind specific HLA allelic variants of interest. Nastke et al. used two predictive algorithms to assess all 42,838 possible HHV-6B nonamers for predicted binding affinity to DRB1*0101 and selected 322 candidates, of which 12 were confirmed as CD4+ T-cell epitopes (51). More recently, Martin et al. took a similar approach aimed at CD8+ T-cell epitopes restricted by HLA-B*0801 and confirmed 16 epitopes by cytolytic functional assays (109). Another study prefaced epitope prediction with a preliminary assay step, by first separating HHV-6 proteins into molecular weight fractions from either HHV-6B virions or infected cell lysates, and testing these fractions for PBMC IFNγ responses (107). Fractions eliciting significant cytokine response were analyzed by mass spectrometry to identify candidate HHV-6B antigenic proteins. Within these, the top 463 predicted HLA DR1-restricted epitopes were then tested using synthetic peptides and 107 were confirmed as epitopes.

Martin et al. used a hybrid of the two approaches, focusing on predicted HLA-A*0201 epitopes within HHV-6B proteins U11, U54, and U90 (homologs of HCMV antigens pp150, pp65, and IE1, respectively) (104). To enrich virus-specific CD8+ T cells, PBMCs were incubated with 12 HHV-6B peptides of interest for 10–14 days, and then re-stimulated with peptide-loaded CD40-activated autologous B cells and IL-2. This study showed CD8+ T-cell clones restricted to three peptides derived from U11 or U54 could recognize HHV-6B-infected cells and produce IFNγ, TNFα, and granzyme B. A summary of these studies is in Table 2.

**Table 2:** Summary of published studies of HHV-6 T-cell antigens.

Study	Approach	Methods	CD4 T-cell antigens confirmed	CD8 T-cell antigens confirmed
Martin et al. (104)	CMV homolog selection and epitope prediction (HLA-A*0201)	ELISA, multimer staining, cytotoxicity assay	(NA)	U11, U54

Nastke et al. (51)	Computer-based epitope prediction (DRB1*0101)	Cytokine bead assay, intracellular cytokine secretion (ICS), ELISpot, HLA-peptide tetramer staining	U11, U14, U38, U48, U54, U47	(NA)

Gerdemann et al. (40)	CMV homolog selection	ELISpot, ICS, cytotoxicity assay	(NA)	U11, U14, U54, U71, U90

Iampietro et al. (115)	CMV homolog selection	ICS, ELISA, cytotoxicity assay	(NA)	U54

Becerra-Artiles et al. (107)	Selection by antigenic gel fractions of HHV-6B proteins followed by computer-based epitope prediction (DRB1*0101)	ELISA, ELISpot, mass spectrometry, SDS-PAGE, fluorescence-polarization HLA peptide-binding competition assay	U11, U14, U31, U39, U41, U48, U54, U57, U90, U100	(NA)

Halawi et al. (105)	CMV homolog selection	ICS, ELISpot, ELISA	(NA)	U11, U90

Martin et al. (109)	Computer-based epitope prediction (HLA-B*0801)	ELISA, ELISpot, cytotoxicity assay, HLA-peptide multimer staining	(NA)	U3, U7, U26, U29, U31, U38, U41, U42, U53, U59, U64, U72, U79, U84, U86, B4

## A High-Throughput Approach to T-Cell Epitope Discovery in HHV-6B

The above approaches have limitations; use of selected ORFs leaves most HHV-6 proteins unexplored while algorithms are imperfect and do not address the full range of possible HLA restrictions. We are thus left with an incomplete view of T-cell specificity for this virus, which could be improved by an approach covering all HHV-6 proteins and diverse HLA haplotypes. Our lab has developed a high throughput, HLA-agnostic method to characterize T-cell immunity to large-genome viruses. Donor PBMCs are stimulated with whole virus, and activated T cells are sorted by FACS and expanded *in vitro* to produce a polyclonal cell line enriched typically a hundredfold for VSTs above the starting PBMC. Cross-presentation by autologous dendritic cells is used for CD8+ T cells, while addition of UV-inactivated pathogen to PBMC suffices to re-stimulate memory CD4+ responses. Each viral ORF is cloned and expressed *via in vitro* transcription and translation for CD4+ T-cell work or prepared for transfection of COS-7 artificial antigen-presenting cells (APC) for CD8+ T-cell studies. The bulk expanded T cells are then assayed with suitable APC and each individual viral protein, as documented for vaccinia, HSV-1, HSV-2, VZV, and *Mycobacterium tuberculosis* (110–114). This approach is now being applied in our lab to study CD4+ T-cell responses to HHV-6B and methods are under development to apply it to CD8+ T cells.

## Leading HHV-6 T-Cell Antigens

So far, studies in roseolovirus antigen discovery have investigated HHV-6B. None have explicitly addressed HHV-6A, although cross-reactivity has been described between the two species using bulk expanded cell lines (51) or T-cell clones (108). Using HHV-6B peptides whose HHV-6A homologs differed in one or more amino acid position, Nastke et al. also found that HHV-6A-specific T-cell lines produced IFNγ in response to individual HHV-6B peptides, and *vice versa*, confirming cross-reactivity at the epitope level (51). Two published studies on CD4+ T cells have both focused on DRB1*0101-restricted HHV-6B epitopes (51, 107); the ORF products identified in both of these studies are U11, U14, U48, U54, and U57, most of which are virion proteins. Becerra-Artiles et al. also correlated HLA-DRB1*0101 peptide-binding affinity with the proportion of responding donors and with the magnitude of T-cell responses as measured by ELISpot (107). Studies on CD8+ T cells have been limited to specific ORF products, except for the recent HLA-B*0801-restricted genome-wide screen performed by Martin et al. mentioned above (109). ORF products described by at least two studies as CD8+ T-cell antigens are U11, U54, and U90 (40, 105, 115). Similarly, studies of VSTs for HHV-6 treatment have used U11, U14, and U90 as antigens for creating VST cell lines (86, 87).

Germline integration of a viral genome presents an interesting question: are viral ORFs at the integration site transcribed and translated, and if so, are they targeted by CMI? Integrated HHV-6 genome sequences are divergent from non-integrated HHV-6 genomes but have all genes fully intact, suggesting replication competence (116). Indeed, PBMCs of iciHHV-6A donors can be induced *in vitro* to produce virions, which can then infect non-integrated HHV-6A-negative cells, so, *in vivo* antigen expression from iciHHV-6 ORFs seems plausible (117). Presumably, T cells educated in thymi of people with iciHHV-6 would be tolerized to HHV-6 antigens; however, iciHHV-6 individuals actually have high frequencies of CD8+ T cells specific for products of U54 (118) and U90 (3), suggesting active immune surveillance. More research is needed to compare the T-cell response to HHV-6 in people with and without chromosomal integration.

## Future Directions

Research on HHV-6 T-cell epitopes to date has largely shown a selection bias toward viral proteins that are homologs of known HCMV antigenic proteins, and it is not clear where these fit into the overall pattern of immunodominance and population prevalence of T-cell epitopes for this virus. This field is entering a new phase of using high-throughput methods to solve this question. Other methods similar to the one described above would also be useful and informative. For example, instead of using *in vitro* expressed proteins to screen for viral antigens, one could use peptide mixes for each protein, as has been done for HCMV and HHV-8. This process could be leveraged by focusing on peptides predicted to bind any of a given donor’s HLA alleles; this would reduce cost, making it feasible to study many more donors and identify antigens and epitopes immunoprevalent across a wide range of HLA alleles.

Such approaches will provide a much fuller understanding of HHV-6 T-cell epitopes, which will in turn inform development of treatment modalities for immunocompromised patients suffering from reactivation. VST immunotherapy for SCT recipients is a nascent field that has garnered much interest in recent years as a promising alternative to antiviral drugs that have associated toxicities or are altogether ineffective (particularly for adenovirus and BK virus) (3, 40, 86, 87, 119, 120). Results of early clinical trials are promising but still anecdotal. Deeper knowledge of T cell epitopes and antigens could help optimize VST products for broad applicability across HLA alleles. In addition, since HHV-6B infection has been associated with various autoimmune disorders (48, 89, 121) and drug reaction with eosinophilia and systemic symptoms (DRESS) (122–124), VSTs lacking specificity for HHV-6 epitopes implicated in these disorders could be preferable. Finally, although a vaccine for HHV-6B is not a current public health priority, future development may benefit from thorough knowledge of HHV-6B T-cell epitopes gained from ongoing research.

## Author Contributions

All authors wrote and edited the manuscript.

## Conflict of Interest Statement

JH has served as a consultant for Chimerix, Inc. and Nohla Therapeutics, Inc. and has received research support from Chimerix, Inc. and Shire. The remaining authors declare that the research was conducted in the absence of any commercial or financial relationships that could be construed as a potential conflict of interest.
